# Isolation, Identification of Three Prometryn-Degrading Strains and Their Synthetic Consortium: Degradation Characteristics and Soil Remediation Potential

**DOI:** 10.3390/microorganisms14092057

**Published:** 2026-09-15

**Authors:** Dong Liang, Wenru Wang, Yuhao Wang, Shuangcai Xiao, Yu Hao, Yutong Song, Lirui Sun, Qingmin Kong, Jiaying Xin

**Affiliations:** 1 College of Food Engineering, Harbin University of Commerce, Harbin 150028, China; liangdong199082@163.com (D.L.); 17713345453@163.com (W.W.); 18434260612@163.com (Y.W.); xiaoshuangcai03@163.com (S.X.); lesliehuc2020@163.com (Y.S.); fere_sun@163.com (L.S.); 2 Tourism & Cuisine College, Harbin University of Commerce, Harbin 150028, China; haoxiaoyu1994@163.com

**Keywords:** prometryn, synthetic consortium, *Stenotrophomonas*, soil enzyme activity, maize detoxification, phytotoxicit

## Abstract

Prometryn, a methylthio-s-triazine herbicide, persists widely in agricultural soils after long-term application, causing crop phytotoxicity and potential human health risks, and microbial degradation offers an eco-friendly, cost-effective strategy for remediation. In this study, three prometryn-degrading bacterial strains were isolated from long-term contaminated cornfield soil in Harbin, China, and identified as *Pseudomonas* sp. ZM-1, *Achromobacter* sp. ZM-2 and *Stenotrophomonas* sp. ZM-3 based on morphological, biochemical and 16S rDNA sequence analysis; strain ZM-3 is, to the best of our knowledge, the first reported pure-culture *Stenotrophomonas* isolate with confirmed prometryn-degrading capability. The three strains degraded 97.3%, 85.1% and 92.8% of 100 mg·L^−1^ prometryn within 48 h respectively. The 1:1:1 synthetic consortium removed 96.8% of prometryn in 15 h, showing markedly superior degradation efficiency compared with single strains, and its 12-h degradation rate reached 96.87% with a half-life of 2.2 h following response surface optimization. In soil microcosms spiked with 20 mg·kg^−1^ prometryn, the consortium reduced the pollutant half-life from 58.2 days to 7.8 days and achieved 96.3% removal after 30 days, alongside enhanced soil dehydrogenase, catalase and urease activities. High-throughput 16S rRNA gene sequencing revealed directional succession of the soil bacterial community, enrichment of core degrading taxa, and predicted enrichment of xenobiotic biodegradation pathways during remediation. Maize pot experiments confirmed that the consortium significantly alleviated prometryn phytotoxicity at the tested concentration, restoring plant growth parameters to 95–97% of the uncontaminated control. This study provides an efficient synthetic microbial consortium for bioremediation of prometryn-contaminated agricultural soils.

## 1. Introduction

Prometryn, a selective methylthiotriazine herbicide, acts by inhibiting photosystem II electron transport [1]. Its broad spectrum, good selectivity, and long persistence make it a common choice for controlling annual grasses and broadleaf weeds in corn, cotton, rice, and soybean fields [2]. The same chemical stability that ensures field efficacy, however, creates prolonged environmental persistence. With low water solubility (approximately 33 mg·L^−1^ at 20 °C) and strong soil adsorption, prometryn was reported to have half-lives of 274–361 days in soil, 28 days in freshwater, and 55–75 days in seawater [3]. Large-scale field surveys in Northeast China, the main maize-producing region, have shown that long-term repeated application has led to widespread prometryn residues in agricultural soils, with detection rates exceeding 40% in typical corn-growing areas, accompanied by phytotoxicity to rotational crops and potential groundwater leaching risks [4,5]. Prometryn was also identified as an endocrine disruptor capable of inducing oxidative stress in non-target aquatic and soil organisms even at environmentally relevant concentrations [6]. Dietary intake of contaminated farm produce is the primary human exposure route of prometryn, and cumulative exposure to s-triazine herbicides carries potential carcinogenic and reproductive risks [7]. Prometryn was classified as a potential carcinogen by the United States Environmental Protection Agency and banned in the European Union in 2004, and microbial degradation served as a cost-effective and sustainable remediation approach for polluted environments.

Microbial degradation offers a cost-effective and ecologically sustainable approach for herbicide residue removal [1]. Several prometryn-degrading bacterial strains have been documented to utilize this herbicide as a carbon, nitrogen, or energy source and enzymatically convert it into less toxic or non-toxic metabolites; among these, the halotolerant strain *Paenarthrobacter ureafaciens* PC is capable of utilizing prometryn as its sole carbon and nitrogen source. This strain completely degraded 20.00 mg/L prometryn within 12 h under saline conditions (30.0 g/L NaCl), and its genome sequencing revealed key genes potentially involved in the degradation pathway [8]. These studies provide strain resources and a theoretical basis for prometryn biodegradation.

However, most relevant studies have focused on pure cultures, and single strains exhibit inherent practical limitations owing to their narrow environmental adaptability. Their degradation activity is strongly influenced by soil temperature, pH, moisture, nutrient availability, and competition with indigenous microorganisms, which commonly results in poor colonization efficiency and an inability to rapidly establish functional degrading populations in complex soil matrices. Their degradation pathways were often incomplete and caused toxic intermediate accumulation and secondary pollution. Recent work indicated that prometryn frequently co-occurred with other herbicides such as acetochlor in agricultural soils, and such co-contamination altered the community structure of key bacterial genera including Sphingomonas and Nocardioides [9] and produced antagonistic effects that significantly suppressed prometryn degradation. These observations collectively demonstrated the severe limitations of single-strain strategies in realistic complex soil remediation.

Synthetic microbial consortia represent a promising alternative strategy: assembling strains with complementary metabolic capabilities enables metabolic division of labor and synergistic effects, thereby enhancing degradation efficiency, broadening environmental adaptability, and improving system robustness [10]. The synthetic consortium L1, developed for sulfonylurea herbicide degradation, has demonstrated that rare species maintain microbial network stability and that positive interspecies interactions are strengthened with increasing substrate diversity [11]. Mixed cultures consistently outperform single strains in the degradation of s-triazine herbicides. For instance, immobilization of a prometryn-degrading consortium in a continuously operated biofilm reactor achieved 100% removal efficiency at a volumetric removal rate exceeding 20 g·m^−3^·h^−1^, far outperforming conventional batch pure culture systems. This body of work provides a solid theoretical and practical foundation for the application of synthetic microbial consortia in the remediation of prometryn pollution.

Despite these advances, three critical knowledge gaps persist that constrain the development of practical bioaugmentation strategies for prometryn-contaminated soils. First, most existing studies focus on single-strain isolates that typically exhibit narrow environmental adaptability, incomplete degradation pathways, and poor colonization capacity in complex soil matrices, with toxic intermediate accumulation and weak competitiveness against indigenous microorganisms further limiting their in situ remediation performance under field conditions. Second, no synthetic consortium composed of *Pseudomonas*, *Achromobacter* and *Stenotrophomonas* has been constructed for prometryn degradation to date. Although *Stenotrophomonas* species are well documented for their metabolic versatility and high stress tolerance and are frequently detected in herbicide-degrading mixed cultures [12], to the best of our knowledge, no pure-culture isolate of this genus with confirmed prometryn-degrading capacity has been reported, leaving the combined degradation potential of these three genera completely unexplored. Third, most studies on prometryn-degrading consortia remain limited to liquid-phase degradation tests, with few having systematically evaluated remediation efficiency, soil ecological function recovery, and crop phytotoxicity alleviation in soil matrices, all critical prerequisites for translating laboratory findings into field applications. To address these gaps, three novel prometryn-degrading strains of the genera *Pseudomonas*, *Achromobacter* and *Stenotrophomonas* were isolated from long-term herbicide-contaminated agricultural soil in this study. Following systematic comparison of their growth characteristics, environmental tolerance, and degradation kinetics, a metabolically complementary synthetic consortium was constructed and its degradation conditions were optimized via response surface methodology; soil microcosm and maize pot experiments were performed to collectively evaluate prometryn removal efficiency, soil biochemical activity recovery, and phytotoxicity alleviation, providing efficient strain resources and a feasible technical framework for the in situ bioremediation of prometryn-contaminated agricultural soils.

## 2. Materials and Methods

### 2.1. Chemicals and Soil

Prometryn (98% purity) and other s-triazine herbicides (simetryn, ametryn, desmetryn, metribuzin, ≥97% purity) were purchased from Zhejiang Zhongshan Chemical Group Co., Ltd., Huzhou, China and Harbin Limin Agrochemical Technology Ltd., Harbin, China, respectively. Analytical-grade chemicals and HPLC-grade methanol and dichloromethane were used. Soil samples were collected from the top 0–20 cm layer of a cornfield in Zhangjia Village, Harbin, China, with five years of prometryn application. A total of 300 samples were sieved (20-mesh), sealed and stored at 4 °C. The soil was classified as sandy loam, with a pH of 6.8 ± 0.2 (determined in a 1:2.5 *w*/*v* water suspension), an electrical conductivity of 0.41 ± 0.04 mS·cm^−1^, 2.30 ± 0.21 g·kg^−1^ organic matter, 1.12 ± 0.13 g·kg^−1^ total nitrogen, 0.038 ± 0.005 g·kg^−1^ available phosphorus, 127 ± 11 mg·kg^−1^ available potassium, and a cation exchange capacity of 15.6 ± 1.4 cmol·kg^−1^. These parameters are consistent with the typical characteristics of maize field soil in the southern Songnen Plain. Uncontaminated soil with similar texture and physicochemical properties, collected from an adjacent fallow field within the same geographic region, was used for the microcosm and pot experiments.

### 2.2. Enrichment, Isolation and Purification

Enrichment and isolation were done as described earlier [9]. We added 10 g of contaminated soil to 100 mL of basal salt medium (BSM) with 100 mg·L^−1^ prometryn as the only carbon source. The BSM contained (per liter) NH_4_NO_3_ 1.0 g, MgSO_4_·7H_2_O 0.2 g, K_2_HPO_4_ 1.0 g, KH_2_PO_4_ 1.0 g, NaCl 0.2 g, and 1 mL of trace element solution, pH 7.0. The culture was shaken at 30 °C and 160 rpm for 7 days. Then 5% (*v*/*v*) of this culture went into fresh BSM with the same prometryn concentration, and we repeated this transfer three times. The final enrichment was diluted in series from 10^−3^ to 10^−7^ and spread on LB agar plates. After 2–3 days at 30 °C, single colonies with different shapes were picked and restreaked on LB plates repeatedly until pure cultures came out.

To test degradation, we took 1 mL of culture, spun it at 12,000× *g* for 10 min, and extracted the supernatant with an equal volume of dichloromethane. The extract was dried under nitrogen and redissolved in methanol. HPLC ran on a C18 column (4.6 × 250 mm, 5 μm) at 25 °C, with methanol/water (80:20, *v*/*v*) as the mobile phase at 1.0 mL/min. Prometryn was measured at 216 nm. Based on the degradation performance of each isolate against 100 mg·L^−1^ prometryn in BSM, three highly efficient degraders were selected and designated as ZM-1, ZM-2 and ZM-3.

The three strains were identified via 16S rRNA gene sequencing. Genomic DNA was extracted using a bacterial DNA kit (CW0552, Cowin Biotech, Taizhou, China), and the 16S rRNA gene was amplified with universal primers 27F (AGAGTTTGATCCTGGCTCAG) and 1492R (GGTTACCTTGTTACGACTT). Purified PCR products were sequenced by Sangon Biotech (Shanghai, China), and the resulting sequences were aligned against the NCBI database using BLAST+2.15.0 and deposited in GenBank under accession numbers PV643981 (ZM-1), PV643982 (ZM-2), and PZ433558 (ZM-3).

### 2.3. Identification of Strains

#### 2.3.1. Morphological and Physiological/Biochemical Tests

Following observation of colony morphology (shape, size, color, edge, and surface texture) and Gram staining for all strains, physiological and biochemical tests were performed in accordance with Bergey’s Manual of Determinative Bacteriology [13], with full assay panels covering oxidase, catalase, methyl red, Voges-Proskauer, citrate utilization, nitrate reduction, starch hydrolysis, gelatin liquefaction and various sugar fermentation for strains ZM-1 and ZM-2, and only selected tests for strain ZM-3.

#### 2.3.2. 16S rDNA Sequencing and Phylogenetic Analysis

We extracted genomic DNA using a bacterial DNA kit (CW0552, Cowin Biotech). The 16S rDNA gene was amplified with the universal primers 27F and 1492R, following the universal 16S rRNA gene amplification protocol widely used in environmental microbiology research [14]. PCR amplification was carried out with an initial denaturation at 94 °C for 5 min, 30 cycles of 94 °C for 30 s, 55 °C for 30 s, and 72 °C for 1.5 min, and a final extension at 72 °C for 10 min; the purified products were sequenced by Sangon Biotech (Shanghai, China), yielding nearly full-length 16S rDNA sequences of approximately 1400 bp for all three strains.

**Figure 1:**
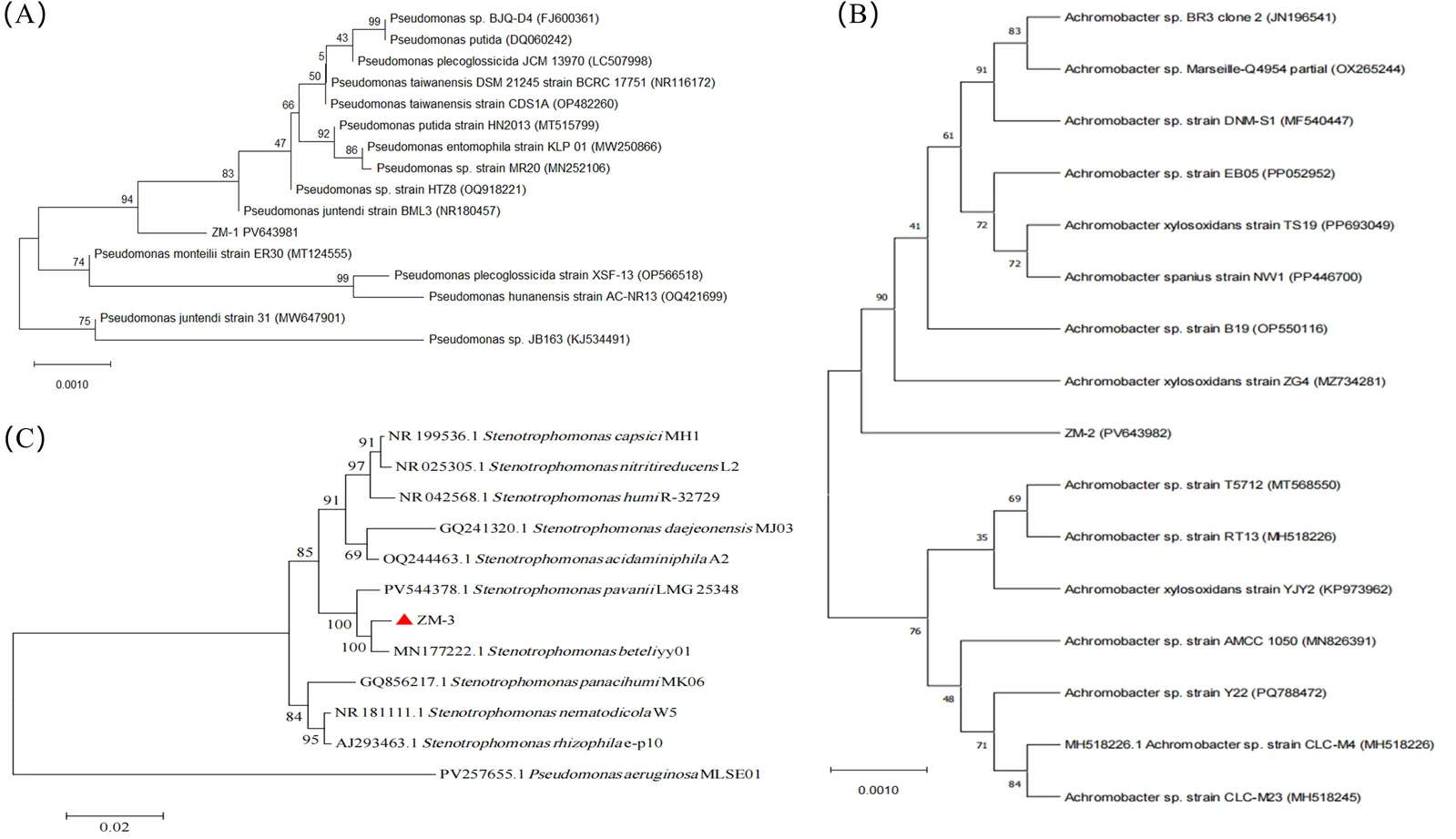
Phylogenetic trees based on 16S rRNA gene sequences showing the phylogenetic positions of the three degrading strains: (**A**) strain ZM-1; (**B**) strain ZM-2; (**C**) strain ZM-3.

BLAST analysis revealed 99.6% similarity of ZM-1 to *Pseudomonas monteilii*, 99.8% for ZM-2 to *Achromobacter xylosoxidans*, and 99.5% for ZM-3 to *Stenotrophomonas maltophilia*, based on which a phylogenetic tree was constructed using MEGA X with the neighbor-joining method and 1000 bootstrap replicates [15]. The sequences have been deposited in GenBank under accession numbers PV643981 (ZM-1), PV643982 (ZM-2), and PZ433558 (ZM-3) (Figure 1). The phylogenetic tree placed ZM-3 within the genus *Stenotrophomonas*, where it formed a clade with *S. capsici* MH1, *S. nitritireducens* L2, *S. humi* R-32729, *S. daejeonensis* MJ03, *S. acidaminiphila* A2, *S. pavani* LMG 25348, *S. betelivy* 01, *S. panacium* MK06, *S. nematodicola* W5, and *S. rhizophila* e-p10. *Pseudomonas aeruginosa* MLSE01 was used as an outgroup.

### 2.4. Growth and Degradation Assays

Cell suspensions of each strain were prepared at OD_600_ = 1.0 (approximately 1 × 10^8^ CFU·mL^−1^), and 5% (*v*/*v*) was inoculated into BSM without prometryn for growth curves, with incubation at 30 °C and 160 rpm for 48 h and OD_600_ measured every 4 h in triplicate; for degradation tests, BSM was spiked with prometryn to 100 mg·L^−1^ and inoculated with the same 5% inoculum, while uninoculated controls were included to assess abiotic degradation. Samples were taken at 3, 6, 9, 12, 15, 18, 24, 36, and 48 h, extracted with an equal volume of dichloromethane, dried under nitrogen, redissolved in methanol, filtered through a 0.22 μm filter, and analyzed on an Agilent 1260 HPLC equipped with a C18 column (4.6 × 250 mm, 5 μm) using methanol-water (80:20, *v*/*v*) at 1 mL·min^−1^, with the column maintained at 25 °C and detection at 216 nm [2]. The analytical method was validated prior to sample analysis. For prometryn determination, the linear range in liquid medium was 0.5–200 mg·L^−1^ (R^2^ > 0.999) with limits of detection (LOD, S/N = 3) and quantification (LOQ, S/N = 10) of 0.02 and 0.06 mg·L^−1^, respectively; for soil samples, average recoveries at three spiked levels ranged from 89.2% to 94.7% with relative standard deviations (RSDs) below 5%, and the method LOD was 0.01 mg·kg^−1^.

Prometryn concentrations were determined from a standard curve, degradation rates calculated as (C_0_ − C_t_)/C_0_ × 100, and the degradation time course fitted to a first-order kinetics model: C_t_ = C_0_ e^−kt^. And the half-life came from t_1/2_ = ln2/k [16].

### 2.5. Single-Factor Experiments

To define the environmental tolerance ranges of the degrading strains and underpin their field application, we systematically evaluated the effects of temperature, pH, and inoculum size on the growth and degradation performance of the three pure strains and the synthetic consortium in BSM. This multifactorial design was conducted to determine optimal degradation conditions for subsequent large-scale inoculum production and immobilized agent formulation, as well as to quantify degradation efficiency under fluctuating conditions to predict the field remediation performance of this bioaugmentation strategy across Northeast China and other potential application regions.

We looked at how temperature (20, 25, 30, 35, 40 °C), initial pH (4.0, 5.0, 6.0, 7.0, 8.0, 9.0, 10.0, 11.0, 12.0), and inoculum size (1%, 3%, 5%, 7%, 9%) affect growth (OD_600_ at 48 h) and degradation rate (all strains measured at 48 h). Based on preliminary experiments, single-factor tests were conducted with the remaining two factors fixed at optimal conditions (pH 7.0, 30 °C, 5% inoculum) and all treatments performed in triplicate, consistent with established protocols for triazine-degrading bacteria [17].

### 2.6. Construction of Synthetic Consortium

Each strain was grown to the logarithmic phase, harvested by centrifugation at 8000 rpm for 5 min, washed twice with sterile saline, and resuspended to an OD_600_ of 1.0 (approximately 1 × 10^8^ CFU·mL^−1^).

An equal-volume 1:1:1 ratio was selected as the empirical baseline for consortium construction, representing a widely adopted initial strategy in synthetic microbial consortium studies [17]. This equal-proportion inoculation design was adopted to eliminate bias arising from differences in initial strain abundance and enable unbiased assessment of combined degradation potential and interstrain synergies, serving as a conservative baseline formulation to verify the metabolic complementarity hypothesis without pre-weighting any single strain and aligning with standard protocols for the initial assembly of synthetic degrading consortia reported in previously published work.

Prior to comparative degradation assays, the synthetic consortium suspension was diluted with sterile saline to a final total cell density of approximately 1 × 10^8^ CFU·mL^−1^, matching that of all single-strain treatments to ensure uniform initial cell loads across all groups, with observed differences reflecting compositional effects rather than inoculum size variations.

To compare the synthetic consortium with the single strains, each was inoculated at the same initial cell density into BSM containing 100 mg·L^−1^ prometryn and degradation was tracked over 72 h, as earlier work had shown that constructing synthetic consortia could boost pesticide breakdown [18].

### 2.7. Optimization of Degradation Conditions for the Synthetic Consortium

Single-factor pre-experiments confirmed significant single-peak effects of temperature, pH, and inoculum size on the degradation efficiency of the synthetic consortium, and the factor ranges (28–32 °C, pH 6.5–7.5, 5–7% inoculum) were determined for subsequent response surface optimization to capture factor interactions and locate the global optimum. Based on these results, response surface methodology (RSM) coupled with a Box–Behnken design (BBD), a statistical approach widely adopted for parameter optimization in pesticide biodegradation research [19], was employed to optimize the degradation conditions of the 1:1:1 synthetic consortium, with temperature (A), initial pH (B), and inoculum size (C) as independent variables and the 12-h prometryn degradation rate as the response (Y); each factor set at three coded levels (−1, 0, +1).

A total of 17 experimental runs were designed, including 5 replicates at the central point to estimate pure error. A quadratic polynomial regression model was established to fit the relationship between independent variables and the response value:

(1) $$Y\;=\;\beta_{0}\;+\;\sum_{i\;=\;1}^{3}\beta_{i}X_{i}\;+\;\sum_{i\;=\;1}^{3}\beta_{\mathrm{ii}}X_{i}^{2}+{\;\sum }_{i\;<\;j\;=\;1}^{3}\beta_{\mathrm{ij}}X_{i}X_{j}$$

where Y is the predicted degradation rate, β_0_ is the intercept term, β_i_ is the linear coefficient, β_ii_ is the quadratic coefficient, and β_ij_ is the interaction coefficient.

Analysis of variance (ANOVA) was used to evaluate the significance of the regression model and each factor term. The optimal degradation conditions were predicted by the model, and triplicate validation experiments were conducted to verify the prediction accuracy. The residual prometryn concentration was determined by HPLC as described in Section 2.4.

### 2.8. Soil Microcosm Remediation Experiment

Soil microcosm experiments were performed using artificially contaminated clean soil to evaluate the degradation capacity of the synthetic consortium. Artificially spiked soil was adopted in this study to eliminate interference from residual herbicides, indigenous degrading populations, and complex historical pollution in long-term contaminated soil, so as to accurately quantify the independent degradation contribution of the exogenously inoculated synthetic consortium. This design is a widely accepted standard protocol for the performance evaluation of degrading microbial consortia under controlled conditions. We took uncontaminated soil, dried it to constant weight, and passed 50 g through a 2 mm sieve. Then we spiked the soil with prometryn to a final concentration of 20 mg kg^−1^ to simulate heavily contaminated farmland and evaluate remediation performance under high pollutant load [20]. We took soil samples on days 0, 5, 10, 15, 20, 25, and 30. To measure how much prometryn was left, we extracted each sample with acetone/hexane (1:1) and ran it on HPLC [21].

On day 15, dehydrogenase, catalase, and urease activities were measured using the TTC method, permanganate titration, and the indophenol blue method, respectively, all following the procedures described by Guan et al. [22].

### 2.9. Soil Bacterial Community High-Throughput Sequencing Analysis

Soil samples collected on days 0, 3, 6, 9 and 12 from the soil microcosm experiment were used for bacterial community profiling, with three biological replicates per time point. Total genomic DNA was extracted from 0.5 g fresh soil using the FastDNA^®^ Spin Kit for Soil (MP Biomedicals, Irvine, CA, USA) following the manufacturer’s protocol. The V3–V4 hypervariable region of the bacterial 16S rRNA gene was amplified with primer pairs 338F (5′-ACTCCTACGGGAGGCAGCAG-3′) and 806R (5′-GGACTACHVGGGTWTCTAAT-3′). Triplicate PCR amplicons for each sample were pooled, purified and quantified, then sequenced on an Illumina MiSeq PE300 platform (Illumina, San Diego, CA, USA). Raw reads were quality-filtered with Trimmomatic and merged with FLASH. Chimeric sequences were removed via UCHIME. High-quality clean sequences were clustered into operational taxonomic units (OTUs) at 97% sequence similarity using UPARSE v7.1. Taxonomic annotation was performed against the Silva SSU138 database with the RDP classifier at a 70% confidence threshold. Alpha diversity indices (Shannon, ACE, Chao1) were calculated in Mothur v1.30.2 to evaluate species richness and evenness. Non-metric multidimensional scaling (NMDS) based on Bray–Curtis distances was performed to visualize temporal shifts in bacterial community structure, with permutational multivariate analysis of variance (PERMANOVA) applied to test the significance of structural differences across time points. Linear discriminant analysis effect size (LEfSe) at an LDA score threshold of 2.0 was used to identify stage-specific biomarker taxa, with intergroup comparisons conducted via the Kruskal–Wallis rank sum test at *p* < 0.05 and Benjamini–Hochberg correction for multiple testing; identified biomarkers require cautious interpretation given the limited biological replicates (*n* = 3 per time point). The functional potential of the bacterial community was predicted via PICRUSt2, with annotations mapped to the Kyoto Encyclopedia of Genes and Genomes (KEGG) and Clusters of Orthologous Groups (COG) databases [23]. Correspondence between original sequencing sample IDs and experimental groups is provided in Table A1 of the Appendix A.

Raw 16S rRNA gene sequencing data from this study have been deposited in the NCBI Sequence Read Archive (SRA) under BioProject accession number PRJNA1491339 and SRA study accession number SRP715962.

### 2.10. Pot Experiment with Maize (Detoxification Assay)

Prometryn was first dissolved in methanol and mixed into clean soil to a final concentration of 80 μg·kg^−1^, a realistic level below China’s maximum residue limit for some crops yet still known to harm sensitive plants such as maize, and after the treated soil was air-dried to remove methanol, three treatments were established: (A) a control without prometryn or bacteria, (B) prometryn alone at 80 μg·kg^−1^, and (C) prometryn plus the synthetic consortium (1:1:1 mix, totaling 1 × 10^8^ CFU per kg soil), with six replicates each and 6 kg of soil per pot (23 cm × 16.5 cm × 20 cm).

Maize seeds (*Zea mays* L.) were surface-sterilized, soaked at 45 °C for 4 h, and pre-germinated on sterile gauze at 28 °C for 3 d, after which three germinated seeds were sown per pot. All pots were maintained in a growth chamber at 28 °C under a 16-h light/8-h dark photoperiod (ca. 1000 lx) and watered twice daily with sterile water. After 14 d of cultivation, shoot length, root length, shoot fresh weight, and root fresh weight were measured [2], with mean values from three seedlings per pot used as the statistical unit and each pot treated as one independent biological replicate (*n* = 6 per treatment); soil samples were collected from each pot for residual prometryn quantification.

Two prometryn concentrations were established for distinct experimental objectives: 20 mg·kg^−1^ for the microcosm experiment to assess maximum remediation capacity under high pollutant stress, and 80 μg·kg^−1^ for the pot experiment to evaluate phytotoxicity alleviation at an environmentally relevant residual level. As these concentrations represent distinct contamination scenarios, results from the two assays cannot be directly extrapolated to one another.

### 2.11. Statistical Analysis

All experiments were performed in triplicate (six replicates for the pot experiment), and data are expressed as mean ± standard deviation (SD). Residual normality and variance homogeneity were tested using the Shapiro–Wilk test and Levene’s test, respectively, and single-time-point comparisons were conducted via one-way ANOVA followed by Duncan’s multiple range test. For temporal degradation dynamics with repeated measurements, a repeated-measures ANOVA was applied to assess overall treatment effects and time × treatment interactions. Significance levels were set at *p* < 0.05. All statistical analyses were conducted using SPSS version 26.0 (IBM Corp., Armonk, NY, USA).

## 3. Results

### 3.1. Isolation and Identification of Three Prometryn-Degrading Strains

After enrichment and purification, three strains with strong prometryn-degrading ability were obtained and named ZM-1, ZM-2 and ZM-3. Colony morphologies were as follows: ZM-1 was milky white, round, smooth, with entire margins; ZM-2 was grayish-white, mucoid, and convex; ZM-3 was yellow, irregular, with rhizoid margins. All three strains were Gram-negative rods.

Table 1 summarizes the physiological and biochemical characteristics of strains ZM-1, ZM-2, and ZM-3. For ZM-3, selected tests were performed, and the results are shown in Table 2; its identification was primarily based on 16S rDNA sequencing and morphological features. The 16S rDNA sequences showed that ZM-1 shared 99.6% similarity with *Pseudomonas monteilii*, ZM-2 shared 99.8% similarity with *Achromobacter xylosoxidans*, and ZM-3 shared 99.5% similarity with *Stenotrophomonas maltophilia*. Accordingly, they were identified as *Pseudomonas* sp. ZM-1 (GenBank PV643981), *Achromobacter* sp. ZM-2 (GenBank PV643982), and *Stenotrophomonas* sp. ZM-3 (GenBank PZ433558) (Figure 1).

The phylogenetic tree placed ZM-3 within the genus *Stenotrophomonas*, where it formed a clade with *S. capsici*, *S. nitritireducens*, *S. humi*, *S. daejeonensis*, *S. acidaminiphila*, *S. pavani*, *S. betelivy*, *S. panacium*, *S. nematodicola*, and *S. rhizophila*, with bootstrap values of 91%, 97%, 91%, 69% and 95% where indicated [12]. *Pseudomonas aeruginosa* MLSE01 was used as an outgroup. To the best of our knowledge, this is the first report of a pure-culture *Stenotrophomonas* strain (ZM-3) with confirmed prometryn degradation ability, whereas previous studies have only detected this genus in mixed consortia degrading other s-triazine herbicides [24].

### 3.2. Growth Characteristics and Degradation Characteristics

**Figure 2:**
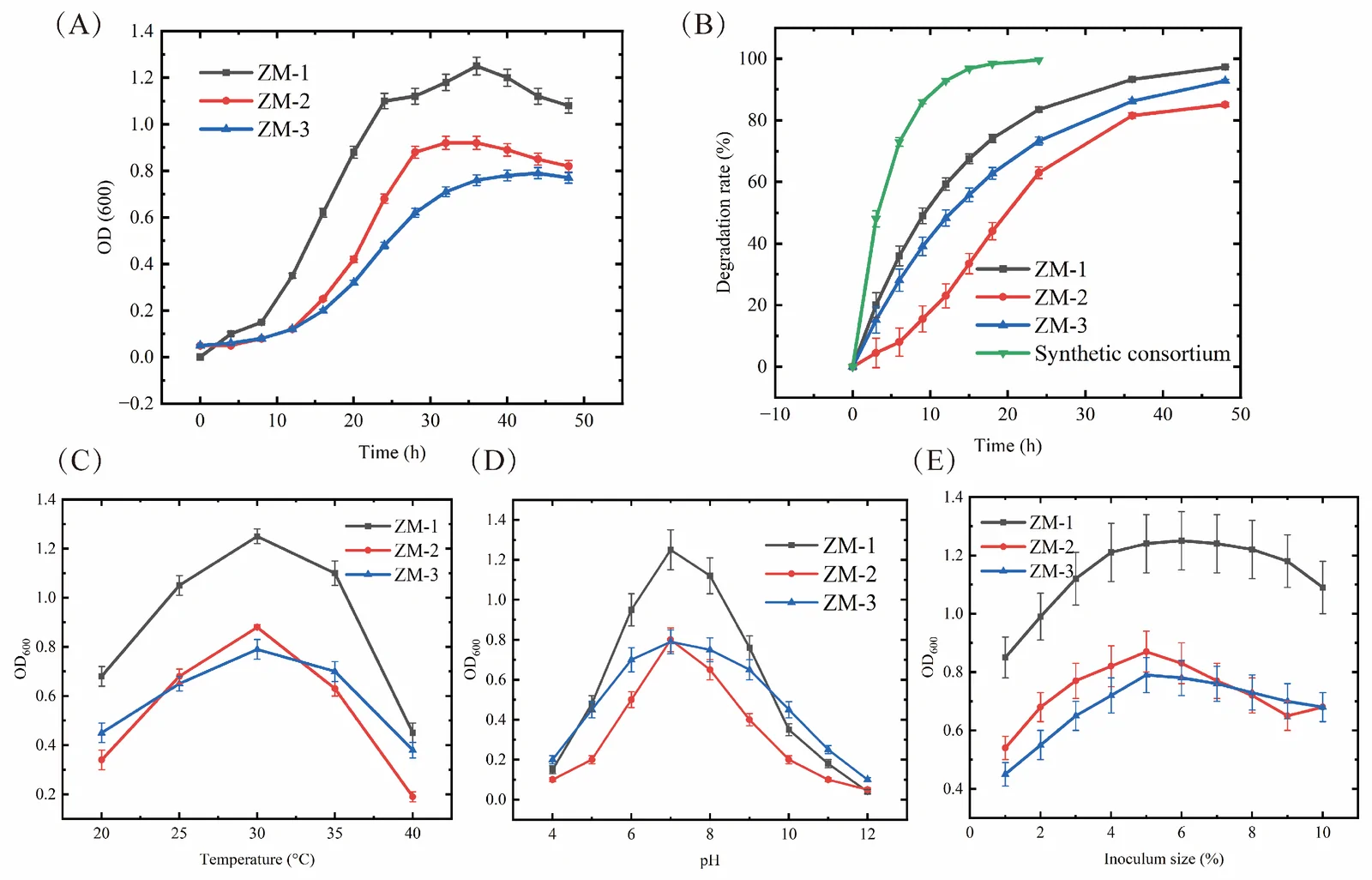
Growth and prometryn degradation characteristics of the three strains under pure culture conditions. (**A**) growth curves of single strains in basal salt medium; (**B**) degradation kinetics of 100 mg·L^−1^ prometryn by single strains and the synthetic consortium; (**C**) effect of temperature on strain growth (OD_600_); (**D**) effect of initial pH on strain growth (OD_600_); (**E**) effect of inoculum size on strain growth (OD_600_). Data are presented as mean ± standard deviation (*n* = 3).

Growth curves (Figure 2A) showed that ZM-2 grew the fastest, reaching stationary phase at 28 h with an OD_600_ of 0.91. ZM-1 peaked at 36 h with an OD_600_ of 0.86. ZM-3 grew more slowly but kept going longer, reaching its highest OD_600_ of 0.79 at 44 h.

Looking at the degradation curves (Figure 2B), ZM-1 broke down 97.3% of prometryn in 48 h (k = 0.075 h^−1^, half-life = 9.2 h). ZM-2 degraded 85.1% in 48 h (k = 0.040 h^−1^, half-life = 17.3 h). ZM-3 degraded 92.8% in 48 h (k = 0.055 h^−1^, half-life = 12.6 h). Notably, the synthetic consortium achieved the fastest and most complete prometryn degradation, removing 96.8% of the target compound within 15 h and substantially outperforming all individual pure strains. This finding aligns with a previous meta-analysis demonstrating that mixed microbial consortia generally exhibit higher s-triazine degradation rates than pure cultures [25]. All three strains utilized prometryn as the sole carbon source, consistent with previous findings that *Pseudomonas* and *Achromobacter* are dominant culturable s-triazine-degrading genera in agricultural soils [26]. Single-factor assays further characterized the effects of temperature, initial pH and inoculum size on strain growth (Figure 2C–E). All three strains reached their maximum biomass at 30 °C and pH 7.0, consistent with their optimal degradation conditions. Strain ZM-3 retained relatively high growth activity at 20 °C, 40 °C, pH 5.0 and pH 9.0, while ZM-1 and ZM-2 showed sharp declines in biomass under these extreme conditions. Biomass of all strains rose with increasing inoculum size and then plateaued, with optimal inoculum levels of 7% for ZM-1, and 5% for both ZM-2 and ZM-3. The growth and degradation parameters of the three strains are summarized in Table 2.

### 3.3. Environmental Tolerance Comparison

To further evaluate the application potential of the three strains, the effects of temperature, initial pH and inoculum size on their growth and degradation performance were investigated.

**Figure 3:**
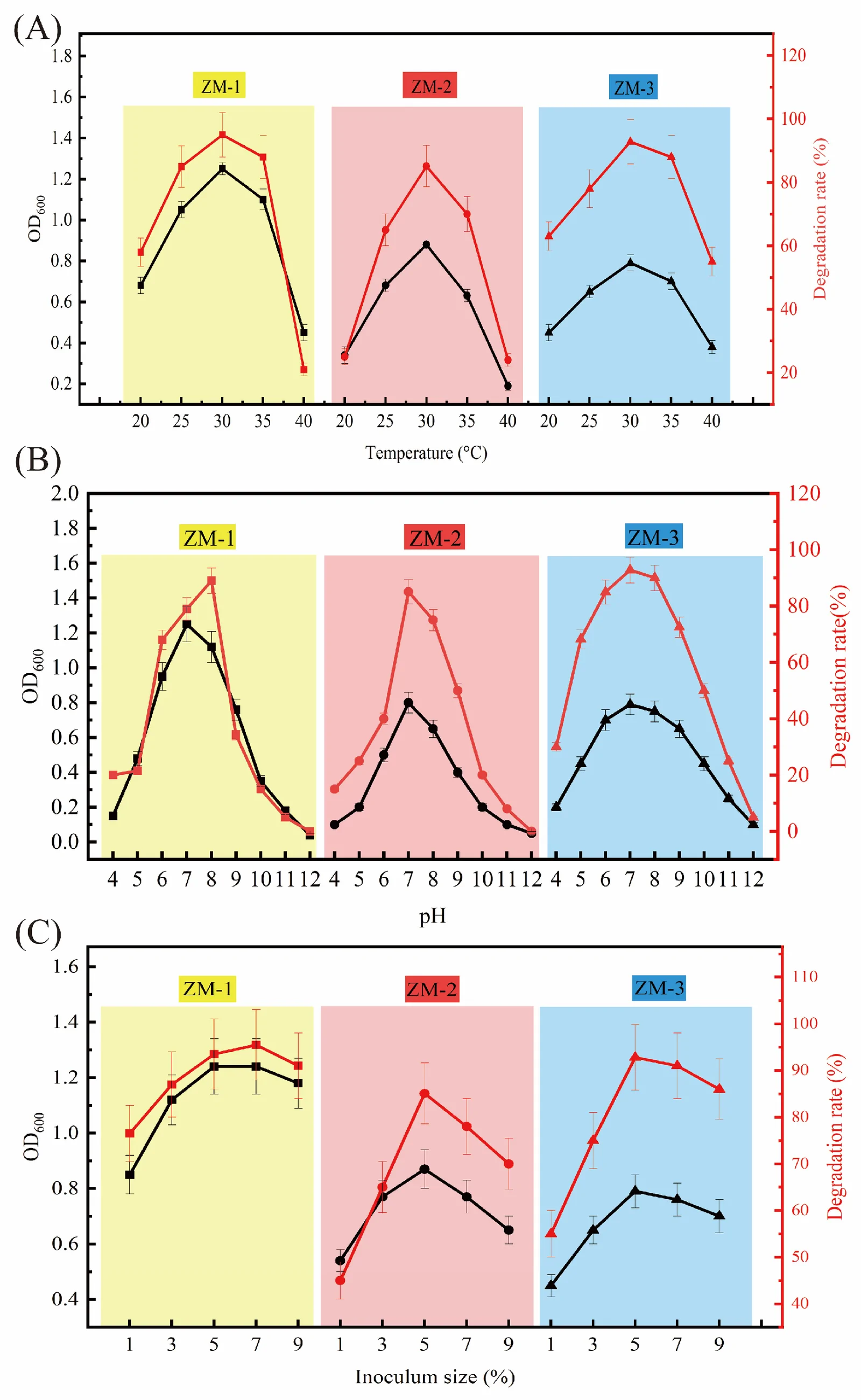
Effects of environmental factors on prometryn degradation efficiency of the three strains. (**A**) Temperature; (**B**) initial pH; (**C**) inoculum size. Data are presented as mean ± standard deviation (*n* = 3).

Looking at temperature effects first (Figure 3A), all three strains worked best at 30 °C. ZM-3 still managed 58% degradation at 20 °C and 55% at 40 °C. In contrast, strain ZM-1 retained only 21% of its maximum degradation efficiency at 40 °C, compared with 24% for ZM-2. Regarding pH effects (Figure 3B), ZM-3 maintained 68.3% and 72.5% degradation at pH 5.0 and pH 9.0, respectively, whereas ZM-1 achieved only 21.5% and 34.2% under the same conditions, demonstrating that ZM-3 had a substantially broader tolerance to both pH and temperature fluctuations. For inoculum size, the optimal degradation inoculation ratios were 7% for ZM-1, 5% for ZM-2, and 5–6% for ZM-3 (Table 2). Degradation rates initially rose with increasing inoculum size before plateauing or declining slightly at higher inoculum levels (Figure 3C); this dose–response pattern is characteristic of *Stenotrophomonas* species [17], and the overall degradation performance of ZM-3 is superior to that of most prometryn degraders reported to date.

### 3.4. Optimization of Prometryn Degradation by the Synthetic Consortium

Based on the single-factor test results above, response surface methodology was employed to further optimize the degradation conditions of the synthetic consortium for maximum degradation efficiency.

#### 3.4.1. Regression Model Establishment and Significance Test

The factors and their coded levels for the Box–Behnken design are shown in Table 3.

The BBD experimental design and corresponding degradation rate results are shown in Table 4. Through multiple regression fitting of the experimental data, a quadratic polynomial regression equation of the 12 h prometryn degradation rate (Y) to temperature (A), pH (B), and inoculum size (C) was obtained in coded values:

(2) $$Y\;=\;97.40\;+\;1.46A\;+\;1.65B\;+\;1.21C-0.025\mathrm{AB}\;+\;0.000\mathrm{AC}-0.125\mathrm{BC}-3.13A^{2}-1.40B^{2}-1.82C^{2}$$

The detailed ANOVA results are presented in Table 5. The regression model was highly significant (F = 219.18, *p* < 0.0001), and the lack-of-fit term was not significant (F = 0.86, *p* = 0.5310 > 0.05), indicating that the model had good fitting degree and could accurately predict the degradation efficiency of the synthetic consortium under different conditions.

The determination coefficient R^2^ = 0.9965, adjusted R^2^ = 0.9919, and predicted R^2^ = 0.9745, which were in good agreement with each other. The adequate precision value was 40.43, far greater than the threshold of 4, indicating an adequate signal-to-noise ratio of the model.

Among the three factors, the influence order on degradation rate was pH (B) > temperature (A) > inoculum size (C). Both linear (A, B, C) and quadratic (A^2^, B^2^, C^2^) terms were highly significant (*p* < 0.0001), while all interaction terms (AB, AC, BC) were non-significant (*p* > 0.05), indicating that the three factors exerted predominantly independent effects on degradation efficiency with weak pairwise synergistic interactions.

#### 3.4.2. Interactive Effect Analysis of Factors

**Figure 4:**
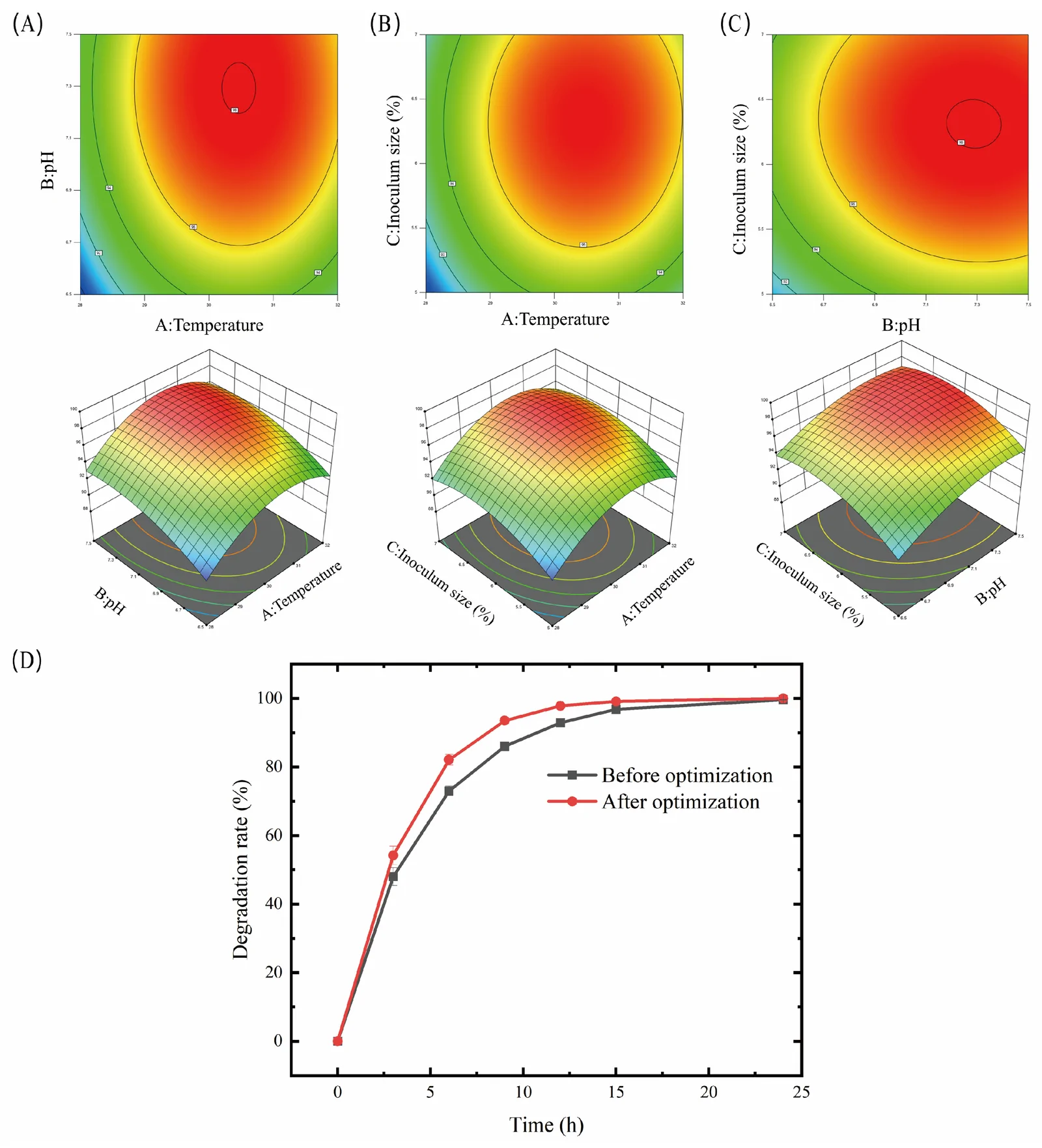
Response surface optimization of prometryn degradation by the synthetic consortium and kinetic validation. (**A**) Contour and 3D response surface plot of temperature × pH interaction; (**B**) contour and 3D response surface plot of temperature × inoculum size interaction; (**C**) contour and 3D response surface plot of pH × inoculum size interaction; (**D**) comparison of prometryn degradation kinetics of the synthetic consortium before and after optimization. Data are presented as mean ± standard deviation (*n* = 3).

The 3D response surface plots (Figure 4A–C) intuitively reflected the pairwise factor interactions on the degradation rate, and consistent with the ANOVA results, the nearly circular contours for all factor pairs confirmed that the interactions between temperature and pH, temperature and inoculum size, as well as pH and inoculum size were not statistically significant, while the degradation rate followed a typical single-peak parabolic trend, increasing initially and then decreasing as each factor increased.

#### 3.4.3. Optimal Condition Prediction and Validation

The model predicted the optimal degradation conditions for the synthetic consortium to be a temperature of 31.73 °C, initial pH of 7.28, and inoculum size of 6.34%, under which the predicted 12-h prometryn degradation rate reached 97.01% with a desirability value of 0.923.

Triplicate validation under the optimized conditions achieved an actual 12-h degradation rate of 96.87 ± 0.15%, closely matching the predicted value and confirming the model’s reliability, while kinetic comparison before and after optimization (Figure 4D) showed that the degradation rate constant k increased from 0.229 to 0.314 h^−1^, the half-life shortened from 3.0 to 2.2 h, the time required to reach 95% degradation decreased from approximately 14 to 10 h, and the overall degradation rate rose by about 30%. The 12-h degradation rate increased from 92.9% to 97.8%, showing a significant optimization effect. The results demonstrated that response surface methodology effectively optimized the environmental degradation conditions and unlocked the degradation potential of the consortium under liquid-culture conditions and improved the degradation efficiency.

### 3.5. Soil Microcosm Remediation

**Figure 5:**
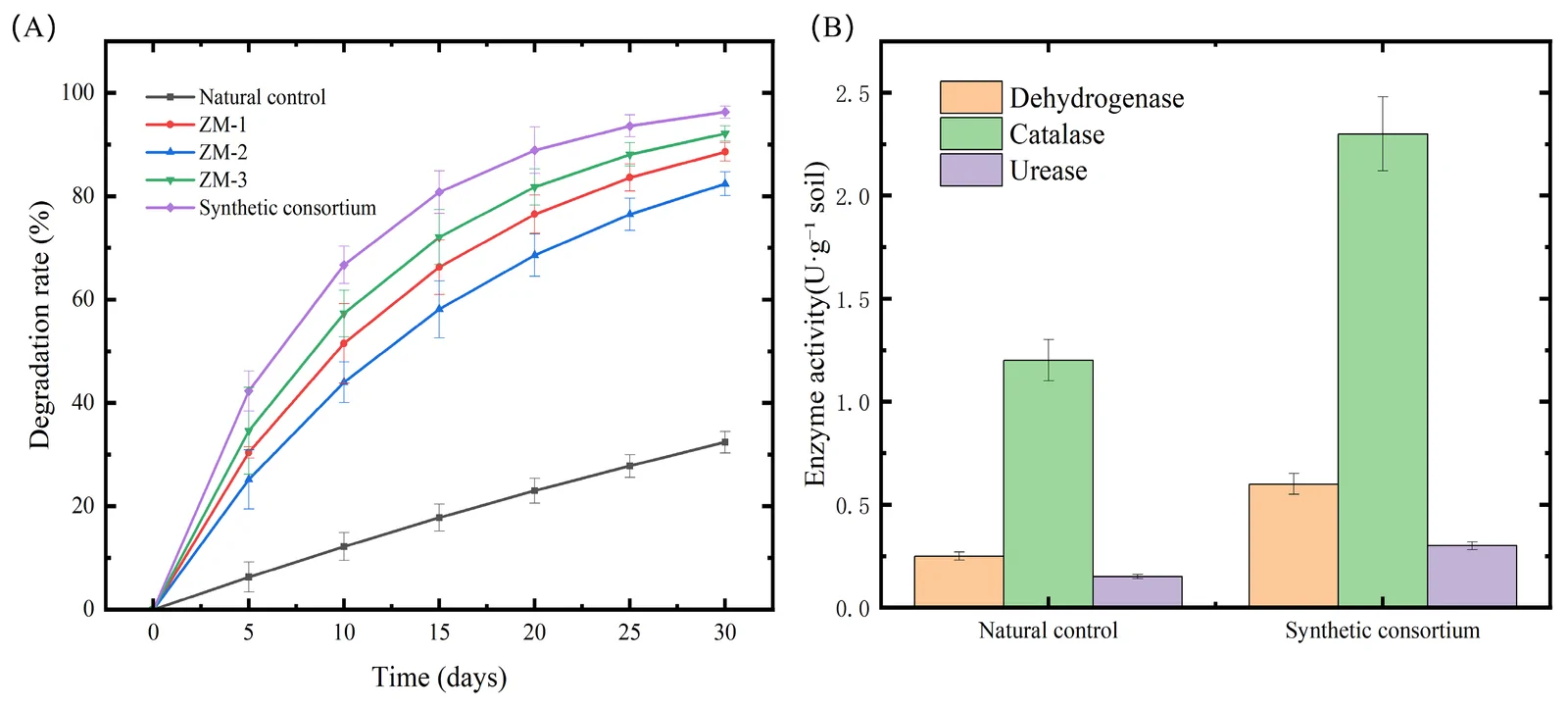
Remediation effect and soil enzyme activity recovery in prometryn-contaminated soil. (**A**) Degradation dynamics of 20 mg·kg^−1^ prometryn in soil microcosms inoculated with single strains and the synthetic consortium within 30 days; (**B**) soil dehydrogenase, catalase and urease activities after 15 days of remediation. Data are presented as mean ± standard deviation (*n* = 3).

On the basis of liquid-culture optimization, the remediation effect of the synthetic consortium on prometryn-contaminated soil was further verified through soil microcosm experiments (Figure 5A). Although the synthetic consortium showed excellent degradation performance in liquid medium, its actual remediation effect in complex soil environment needs further verification.

In soil with 20 mg·kg^−1^ of prometryn, the natural control broke down very slowly. Its half-life was 58.2 days, and only 32.4% ± 2.1% was gone after 30 days. Adding single strains speeded things up, with ZM-3 working the best—half-life dropped to 9.5 days, and 92.1% ± 1.5% was removed. But the synthetic consortium did the best job of all: its half-life was just 7.8 days, and it removed 96.3% ± 1.2% in 30 days, which was significantly better than any single strain (*p* < 0.05). We ran every treatment in triplicate. The first-order kinetic parameters are listed in Table 6. This enhanced degradation efficiency aligns with previous studies demonstrating that mixed microbial consortia outperform single pure strains in soil remediation [10,27].

### 3.6. Soil Enzyme Activities

As shown in Figure 5B, the synthetic consortium significantly enhanced soil enzyme activities relative to the contaminated control after 15 days (*p* < 0.05), with dehydrogenase, catalase, and urease activities exhibiting 2.4-, 1.9-, and 2.0-fold increases, respectively, whereas individual pure strains exerted substantially weaker effects. All enzyme assays were performed in triplicate, and the magnitude of enzyme activity recovery aligned with findings from previous bioremediation studies [27]. Collectively, these findings demonstrate that the synthetic consortium not only achieved efficient prometryn degradation but also promoted the restoration of soil metabolic functionality.

### 3.7. Dynamics of Soil Bacterial Community Structure and Functional Potential During Remediation

#### 3.7.1. Temporal Changes in Alpha Diversity

**Figure 6:**
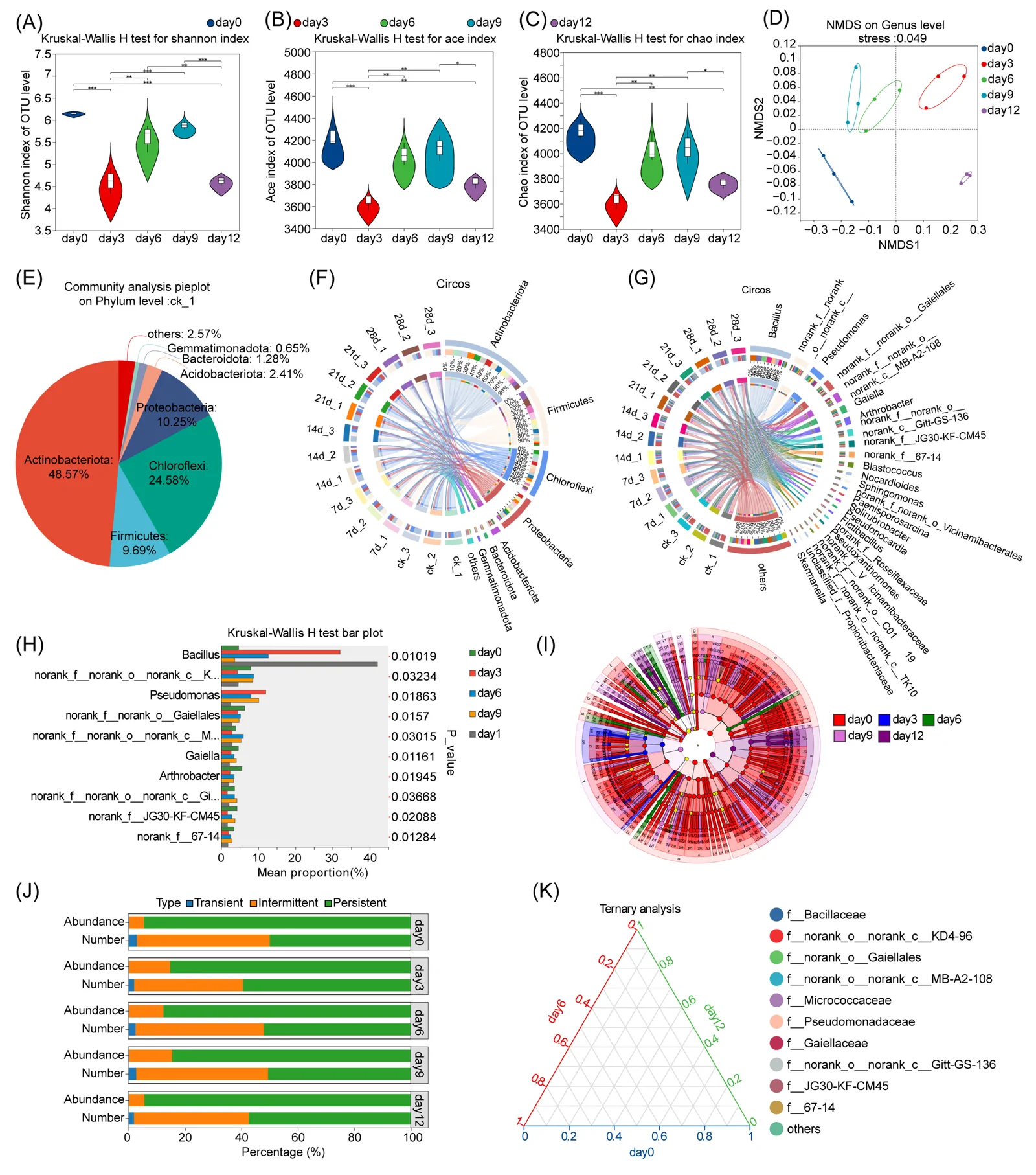
Structural succession of soil bacterial communities during prometryn bioremediation. (**A**–**C**) Bacterial α-diversity indices (Shannon, ACE, and Chao1) across remediation stages. Asterisks denote the significance of pairwise comparisons: * *p* < 0.05, ** *p* < 0.01, *** *p* < 0.001; (**D**) non-metric multidimensional scaling (NMDS) ordination at the genus level; (**E**) relative abundance of dominant phyla in initial soil samples; (**F**,**G**) temporal dynamics of community composition at the phylum and genus levels, respectively; (**H**) genera with significant temporal variation throughout the remediation process; (**I**) LEfSe cladogram illustrating stage-specific biomarker taxa. Full LEfSe outputs with taxonomic labels and LDA-score values are provided in Figure S1 (Supplementary Materials); (**J**) proportions of persistent, intermittent, and transient taxa; (**K**) ternary plot illustrating taxon enrichment preferences.

As shown in Figure 6A–C, soil bacterial α-diversity indices (Shannon, ACE, and Chao1) exhibited consistent temporal dynamics across the full remediation period, featuring an initial significant decline followed by a modest late-stage recovery, with values on day 12 remaining significantly lower than the day 0 baseline (*p* < 0.01).

Untreated initial soil (day 0) exhibited the highest bacterial diversity and richness, with a median Shannon index of approximately 7.5 and mean ACE and Chao1 richness estimators of nearly 4800. Following inoculation with the synthetic consortium, the Shannon index decreased significantly by day 3 (*p* < 0.01), while ACE and Chao1 richness declined continuously to their minimum on day 6. All three indices showed a slight rebound between days 6 and 12, but remained significantly below day 0 levels; the two richness indices fell from approximately 4800 at baseline to approximately 4500 by day 12.

It should be noted that air-drying pretreatment may have altered the initial indigenous bacterial community to some extent. This pattern reflected directional selection of the soil bacterial community during bioaugmentation [28,29]. Early in remediation, strong selective pressure arose from prometryn toxicity and niche competition by the inoculated degrading strains, causing rapid die-off of herbicide-sensitive indigenous bacteria and a sharp drop in diversity and richness. As remediation proceeded, tolerant indigenous functional taxa acclimated to the environment and proliferated during pollutant degradation, driving the mild late-stage rebound in diversity. Nevertheless, persistent pollutant stress and the dominance of functional degraders prevented full recovery to baseline levels, yielding an overall significant reduction in α-diversity.

#### 3.7.2. Structural Differentiation of Bacterial Communities (Beta Diversity)

NMDS at the genus level was performed to assess the overall structural variation of the bacterial community (Figure 6D). The stress value was below 0.2, indicating that the two-dimensional ordination reliably reflected the dissimilarity ranking among samples. Samples across all time points separated clearly along the first axis, forming a distinct temporal gradient with day 0 samples clustering on the left, days 3 and 6 in an intermediate position, and days 9 and 12 shifting to the right; PERMANOVA confirmed significant structural differences in the soil bacterial community across time points (*p* < 0.01), with tight clustering of biological replicates within each time point and markedly greater intergroup than intra-group distances. These findings collectively demonstrate that inoculation with the synthetic consortium drove significant directional succession rather than random fluctuations in the soil bacterial community, a hallmark of effective bioaugmentation-mediated pollutant removal.

#### 3.7.3. Taxonomic Succession of Bacterial Communities

The initial soil bacterial community was dominated by Actinobacteriota (48.57% of total sequences), followed by Chloroflexi (24.58%), Proteobacteria (10.25%), Firmicutes (9.69%), Acidobacteriota (2.41%), Bacteroidota (1.28%), and Gemmatimonadota (0.65%) (Figure 6E). These seven phyla collectively accounted for 97.43% of the total community, with the remaining 2.57% of taxa grouped as others, a phylum-level distribution consistent with the typical bacterial community structure of triazine-contaminated agricultural soils in northern China [30].

The Circos plot in Figure 6F depicts the temporal succession of phylum-level community structure. As remediation progressed, the relative abundances of Proteobacteria and Firmicutes increased progressively, while those of Actinobacteriota and Chloroflexi declined continuously. The enrichment of Proteobacteria reflected the elevated relative abundance of the three inoculated genera, alongside concurrent recruitment of indigenous degrading taxa within this phylum [1].

At the genus level, the Circos plot (Figure 6G) revealed clear directional succession among dominant taxa. The initial soil was dominated by indigenous genera such as Arthrobacter, Gaiella, and norank_f__norank_o__Gaiellales (an unclassified family within the order Gaiellales). Over the course of remediation, genera with documented pollutant-degrading capacities, including *Pseudomonas*, Bacillus, and Pseudoxanthomonas, were significantly enriched and became dominant in the late-stage community. The Kruskal–Wallis H test confirmed significant temporal variation in the relative abundance of 10 key genera including *Bacillus*, *Pseudomonas*, and *Arthrobacter* (all *p* < 0.05, Figure 6H), statistically validating the observed community succession.

#### 3.7.4. Biomarker Taxa and Temporal Survival Strategies

LEfSe analysis identified 38 taxa with LDA scores > 2.0 as stage-specific biomarkers (Figure 6I). Full LEfSe outputs including taxonomic information and corresponding LDA scores are available in Figure S1 of Supplementary Materials. Day 0 biomarkers were mainly affiliated with Actinobacteriota and Chloroflexi, representing the native dominant taxa of the initial soil. Transitional taxa responsive to prometryn stress dominated the biomarker profile from day 3 to day 6, whereas late-stage (day 9–12) biomarkers were concentrated in Pseudomonadaceae, Bacillaceae, and Xanthomonadaceae—three families with well-documented roles in xenobiotic degradation. The LEfSe cladogram further illustrates the hierarchical taxonomic distribution of these biomarkers from phylum to genus level. Such stage-specific biomarker patterns have been widely reported in triazine herbicide bioremediation systems [31].

Bacterial taxa were classified into persistent, intermittent, and transient groups based on their occurrence frequency across all remediation stages (Figure 6J), with persistent and intermittent taxa dominating OTU richness at each stage (49.97–59.47% and 38.51–47.09% of total OTUs, respectively) and transient taxa accounting for only 1.94–2.94%. The proportion of persistent OTUs increased from 49.97% on day 0 to a peak of 59.47% on day 3 and remained at 57.40% on day 12, while transient OTUs declined continuously, reflecting directional selection of core functional taxa under pollutant stress.

Persistent taxa consistently dominated the community in terms of relative abundance, accounting for 84.56–94.53% of total sequences and constituting the functional backbone of the remediation system. Persistent taxa accounted for over 94% of total abundance at both the initial (day 0) and late (day 12) stages, with intermittent taxa rising markedly to 14.61% and 15.22% during the rapid degradation phases (days 3 and 9) alongside a temporary decline in persistent taxa, and transient taxa contributing less than 0.3% of total abundance throughout the process, representing opportunistic groups with negligible functional relevance.

This asymmetric pattern between OTU richness and relative abundance reflects a typical core–periphery community assembly strategy under bioaugmentation. A small subset of persistent core taxa mediated primary pollutant degradation, with intermittent taxa buffering environmental perturbations and supporting auxiliary metabolism during the intensive remediation phase to sustain degradation efficiency and community functional continuity. This assembly pattern is consistent with the directional community succession revealed by α- and β-diversity analyses and previously reported patterns in herbicide-contaminated soils [32].

The ternary plot (Figure 6K) further demonstrates the enrichment preference of key families at three representative stages (day 0, day 6, day 12). Families such as Bacillaceae and Pseudomonadaceae were markedly enriched at day 12, whereas families like norank_o__norank_c__KD4-96 were mainly distributed in the early stage, consistent with the LEfSe and community composition results.

#### 3.7.5. Predicted Functional Potential of the Bacterial Community

**Figure 7:**
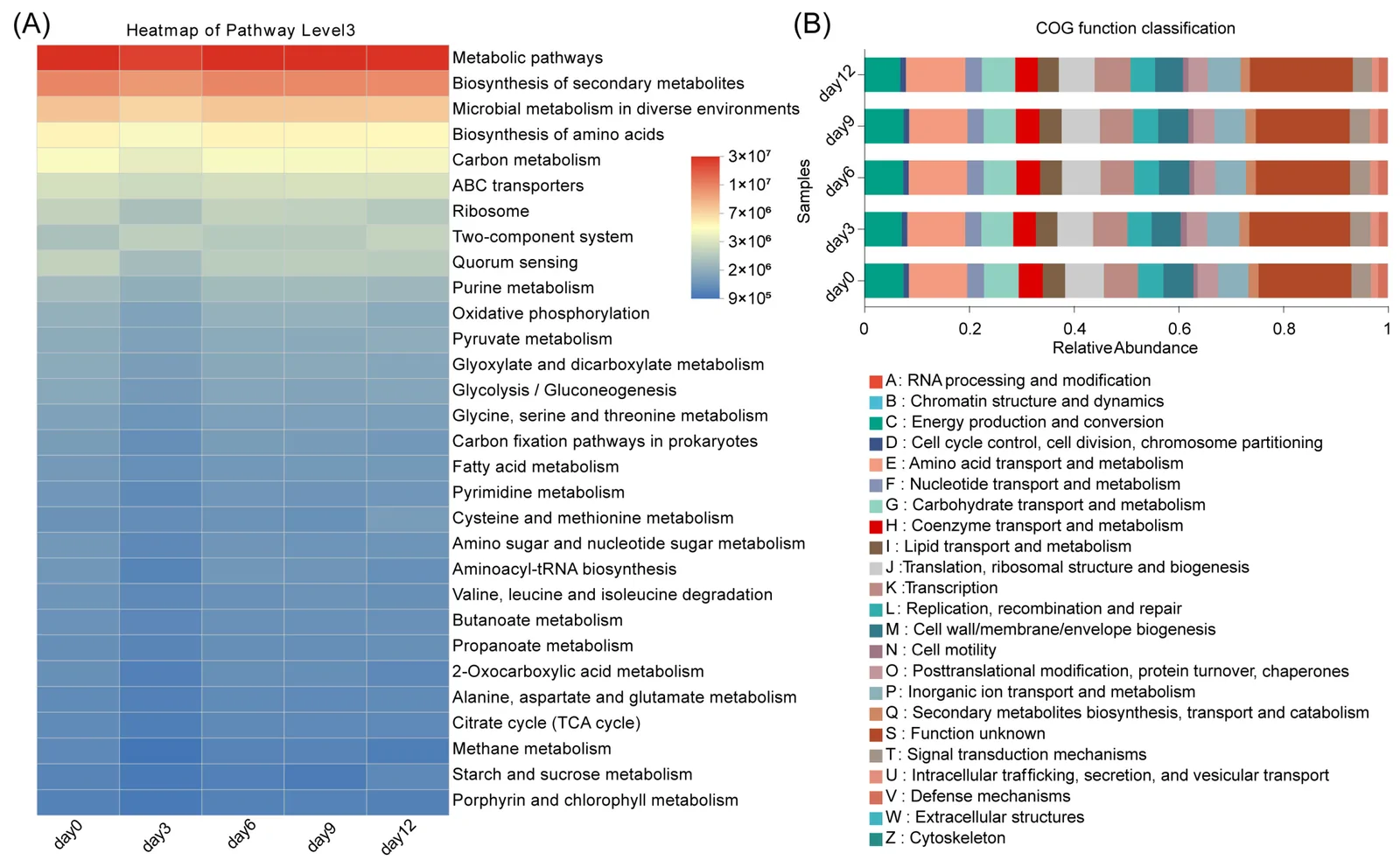
Predicted functional profiles of the bacterial communities at the five sampling stages (day 0, 3, 6, 9, and 12). (**A**) Heatmap of the 30 most abundant KEGG pathways at hierarchy level 3; color intensity denotes predicted pathway abundance, ranging from blue (low) to red (high). (**B**) Stacked bar chart of COG functional classification; each colored segment represents one COG functional category, and its length indicates the relative abundance (0–1, i.e., 0–100%) within each sample.

Functional potential predicted by PICRUSt2 is shown in Figure 7A,B. KEGG pathway analysis revealed that metabolic pathways dominated the functional profile, with carbon metabolism, ABC transporters, biosynthesis of amino acids, ribosome and two-component system ranking as the top five most abundant pathways (Figure 7A). As remediation proceeded, predicted functional potentials related to xenobiotic biodegradation, energy metabolism and membrane transport showed gradual enrichment, which was highly synchronized with the rapid degradation phase of prometryn. Similar predicted enrichment patterns have been reported in multiple pesticide-contaminated soil microbiomes [33].

COG functional classification (Figure 7B) showed that energy production and conversion, amino acid transport and metabolism, transcription, and cell wall/membrane/envelope biogenesis had the highest relative abundances. Notably, functions related to secondary metabolite biosynthesis/transport/catabolism and inorganic ion transport and metabolism increased in the late stage, consistent with the enhanced soil enzyme activities described above. These results indicate that the synthetic consortium not only directly degraded prometryn, but also elevated the overall metabolic activity and pollutant remediation potential of the soil microbial community, which is consistent with metagenomic observations from other triazine bioremediation systems [34], thereby promoting restoration of soil ecological functions.

### 3.8. Maize Detoxification (Pot Experiment)

Table 7 presented maize growth after 14 days under different treatments, showing that in soil contaminated with 80 μg·kg^−1^ prometryn, shoot length, root length, shoot fresh weight, and root fresh weight dropped by 63%, 66%, 76%, and 55%, respectively, compared to the clean control (*p* < 0.01), whereas with the addition of the synthetic consortium, all four parameters recovered to 95–97% of the control values, exhibiting no significant difference from the clean control (*p* > 0.05).

We also measured residual prometryn in pot soils. In the consortium-treated pots, residual prometryn was below the method detection limit of 0.01 mg·kg^−1^, while 68 μg·kg^−1^ remained in the uninoculated contaminated soil. These results demonstrate that the synthetic consortium effectively degraded prometryn and significantly alleviated its phytotoxic effects on maize under the tested conditions, consistent with previously reported findings for *Pseudomonas* sp. DY-1 [2].

## 4. Discussion

This paper reports a *Stenotrophomonas* sp. strain ZM-3 that can efficiently degrade prometryn in pure culture. Although the genus *Stenotrophomonas* has been previously detected in mixed consortia capable of degrading s-triazine herbicides [24], strain ZM-3 represents, to the best of our knowledge, the first pure-culture isolate with confirmed prometryn-degrading capability within the genus *Stenotrophomonas*. Moreover, a synthetic microbial consortium composed of *Pseudomonas* sp. ZM-1, *Achromobacter* sp. ZM-2, and *Stenotrophomonas* sp. ZM-3 shows superior degradation performance and excellent soil remediation effect, effectively alleviating prometryn phytotoxicity to maize.

Strain ZM-3 degrades 92.8% of prometryn within 48 h and maintains high activity across pH 5.0–9.0 and 20–40 °C. Its degradation efficiency and environmental tolerance surpass those of several reported prometryn-degrading bacteria, such as *Pseudomonas* sp. DY-1 [2], *Leucobacter* sp. JW-1 [20], a recently reported *Bacillus* sp. isolate [35], and *Rhodococcus* sp. FJ1117YT [8]. The genus *Stenotrophomonas* is known for its broad environmental adaptability, consistent with its vigorous growth in the rhizosphere and contaminated environments, and its diverse metabolic pathways and strong stress resistance [12], making it an excellent candidate for in situ bioremediation.

In both liquid-culture and soil systems, the degradation performance of this synthetic consortium is significantly superior to that of any single strain. The enhanced performance arises from multiple complementary mechanisms rooted in the differential physiological and metabolic traits of the three strains characterized in pure culture. The staggered growth patterns of the three strains form temporal niche complementarity that sustains continuous and efficient degradation throughout the entire culture cycle: pure-culture growth curves showed that strain ZM-2 enters the logarithmic phase earliest and reaches stationary phase at 28 h, allowing it to rapidly initiate prometryn transformation in the early stage; strain ZM-1 dominates mid-stage degradation with its high specific degradation activity; while the slower-growing but longer-persisting strain ZM-3 maintains degradation function into the late stage, extending the effective degradation window and avoiding intense resource competition that would occur if all strains grew synchronously. Additionally, the divergent environmental tolerance profiles of the strains confer broader adaptability on the consortium. Pure-culture tolerance tests demonstrated that strain ZM-3 retains over 55% degradation activity under extreme conditions (20 °C, 40 °C, pH 5.0, pH 9.0), whereas ZM-1 and ZM-2 lose most of their activity under these conditions. The inclusion of ZM-3 thus provides stress tolerance for the entire consortium, ensuring more stable degradation efficiency under fluctuating environmental conditions, a trait particularly valuable for in-situ soil remediation [12]. Furthermore, metabolic division of labor is hypothesized to enhance the overall degradation depth. Based on well-documented metabolic traits of *Pseudomonas*, *Achromobacter* and *Stenotrophomonas* genera in s-triazine biodegradation [25], we infer that stepwise transformation of prometryn may occur among the three strains, which would alleviate feedback inhibition of intermediates on upstream degrading enzymes and improve overall pathway throughput. It should be explicitly noted that this pathway model remains a literature-based inference and has not been experimentally verified in this study; targeted intermediate detection and genomic functional analysis will be performed in our follow-up work.

Additionally, the consortium may enhance its degradation activity through promoting biofilm formation and quorum sensing [36,37]. Different from single-factor experiments that only evaluate the effect of individual factors, response surface methodology can quantify the influence of factor interactions and obtain the global optimal condition, which further unlocked the degradation potential of the consortium. The optimal temperature and pH ranges determined in this study are highly consistent with the seasonal soil environmental conditions of maize fields in Northeast China during the growing period, which provides a practical basis for the field application of this consortium. The significant recovery of soil dehydrogenase, catalase and urease activities further indicates that the consortium not only efficiently removes prometryn residues, but also restores the ecological metabolic function of contaminated soil, avoiding the secondary ecological damage caused by traditional chemical remediation methods [38].

High-throughput sequencing results provide deeper insights into the micro-ecological mechanisms underlying the remediation process. The significant decline in alpha diversity during the early stage is a typical signature of bioaugmentation in contaminated soil: exogenous degrading strains and prometryn stress jointly filter the indigenous community, favoring tolerant and degrading taxa while suppressing sensitive species [28]. This succession pattern aligns with previous findings on the bioremediation of triazine-contaminated soils. The progressive enrichment of Proteobacteria and Firmicutes reflects the combined action of inoculated strains and indigenous degrading bacteria. The three inoculated genera became significantly enriched in soil concurrent with rapid prometryn transformation, while indigenous degrading genera such as Bacillus and Arthrobacter were concomitantly enriched and likely participated in further transformation of metabolic intermediates. Such metabolic complementarity between exogenous and indigenous microbes constitutes the mechanistic basis for the consortium’s superior degradation performance in soil [39]. This directional community succession may be transient and may cause persistent ecological disturbance to the native soil microbiome. The predicted enrichment of xenobiotic degradation and energy metabolism potentials is consistent with the enhanced enzyme activities, jointly indicating that the consortium drives soil functional improvement through structural and functional remodeling of the bacterial community [34].

Importantly, prometryn phytotoxicity to maize was significantly alleviated at the tested concentration. Although 80 μg·kg^−1^ is an environmentally relevant level below the maximum residue limit for some crops in China, it still caused significant growth inhibition in sensitive crops such as maize [2]. At this concentration, the consortium reduced soil prometryn residues to below the detection limit and restored maize growth to a level comparable to the uncontaminated control. The synthetic consortium reduced soil prometryn residues to below the detection limit, allowing maize to return to normal growth, and given that prometryn residues often inhibited subsequent crops such as maize and soybean, this finding held significant practical value, making the ZM-1 + ZM-2 + ZM-3 consortium a promising bioaugmentation strategy for safe crop production in prometryn-contaminated agricultural fields.

Regarding biosafety, several points need to be clarified. First, strain identification based on 16S rRNA gene sequencing can reliably assign strains to the genus level but has limited resolution for precise species delineation within these three genera; whole-genome sequencing or multilocus sequence analysis will be required for accurate taxonomic identification in future work. Second, 16S rRNA sequence similarity indicated that strains ZM-2 and ZM-3 are phylogenetically close to *Achromobacter xylosoxidans* and *Stenotrophomonas maltophilia*, respectively, both of which include opportunistic pathogenic lineages and may carry antibiotic resistance genes.

Further work is required to elucidate the complete degradation pathway at the genomic level and optimize consortium immobilization carriers to improve field survival stability. Combined application with biostimulation measures such as organic amendment represents another promising direction to further enhance in-situ remediation efficiency [40,41].

## 5. Conclusions

This study isolated three prometryn-degrading bacterial strains belonging to *Pseudomonas*, *Achromobacter* and *Stenotrophomonas* from long-term herbicide-contaminated agricultural soil, constructed a metabolically complementary synthetic consortium, and systematically evaluated its remediation potential in both liquid culture and soil matrices.

Strain ZM-3 is, to the best of our knowledge, the first pure-culture *Stenotrophomonas* isolate with confirmed prometryn-degrading capacity. This finding expands the known taxonomic range of culturable s-triazine-degrading bacteria and provides a novel stress-tolerant strain resource for degrading consortium construction. The three-strain consortium outperformed individual strains in degradation efficiency and environmental adaptability through metabolic complementarity and niche differentiation. This result verifies the feasibility of assembling multi-genus bacterial consortia for enhanced s-triazine remediation, and provides empirical support for the ecological strategy of improving remediation performance via interspecific functional division of labor.

Soil microcosm and maize pot experiments demonstrated that the consortium achieved efficient pollutant removal, restored soil biochemical enzyme activities, alleviated prometryn phytotoxicity and recovered plant growth. The optimal degradation conditions matched well with the seasonal environmental parameters of maize fields in Northeast China, providing a complete practical technical framework from strain screening to condition optimization and effect validation for in situ bioremediation of prometryn-contaminated agricultural soils.

Several limitations of this study should be acknowledged. All experiments were performed under laboratory-controlled conditions with artificially spiked soil, so the long-term in-situ remediation performance and colonization stability of the consortium under actual field conditions remain to be systematically verified. The detailed molecular degradation pathway and actual interstrain metabolic division of labor have not been experimentally confirmed. The 1:1:1 equal-volume ratio used here represents a baseline formulation, and further optimization of strain proportions and cell immobilization strategies is needed to maximize practical remediation efficiency. The limited biological replicates for microbiome analysis mean related ecological conclusions should be interpreted with caution.

Future work will focus on field validation of the consortium, elucidation of degradation mechanisms at the genomic level, and development of immobilized bacterial agents to promote practical application of this bioremediation strategy.

## Figures and Tables

**Table 1:** Physiological and biochemical characteristics of strains ZM-1, ZM-2 and ZM-3.

Test	ZM-1	ZM-2	ZM-3
Oxidase	+	+	−
Catalase	+	+	+
Methyl red	−	−	−
Voges-Proskauer	−	−	−
Starch hydrolysis	−	−	−
Gelatin liquefaction	−	−	+
Nitrate reduction	+	+	−
Citrate utilization	+	+	+
Urease	−	−	−

Note: + positive; − negative.

**Table 2:** Comparison of growth and degradation parameters of the three strains.

Strain	Optimal Temp (°C)	Optimal pH	Optimal Inoculum (%)	Max OD600	Max Degradation Rate (%)	k (h − 1)	t1/2 (h)	R^2^
ZM-1	30	7	7	0.86 (36 h)	97.3 ± 1.2 (48 h)	0.075	9.2	0.978
ZM-2	30	7	5	0.91 (28 h)	85.1 ± 1.5 (48 h)	0.040	17.3	0.956
ZM-3	30–32	6.5–7.5	5–6	0.79 (44 h)	92.8 ± 1.1 (48 h)	0.055	12.6	0.969

**Table 3:** Factors and levels for response surface optimization of the synthetic consortium.

Factor	Symbol	Level −1	Level 0	Level +1
Temperature (°C)	A	28	30	32
Initial pH	B	6.5	7	7.5
Inoculum size (%)	C	5	6	7

**Table 4:** Box–Behnken experimental design and results.

Run	Temperature (°C)	pH	Inoculum Size (%)	12 h Degradation Rate (%)
1	30	6.5	5	91.3
2	30	6.5	7	93.7
3	30	7.5	5	94.9
4	30	7.5	7	96.8
5	28	7	5	89.7
6	28	7	7	92.2
7	32	7	5	92.5
8	32	7	7	95.4
9	28	6.5	6	89.8
10	28	7.5	6	93.1
11	32	6.5	6	92.7
12	32	7.5	6	95.9
13	30	7	6	97.2
14	30	7	6	97.6
15	30	7	6	97.4
16	30	7	6	97.1
17	30	7	6	97.7

**Table 5:** Analysis of variance (ANOVA) for the quadratic regression model.

Source	Sum of Squares	df	Mean Square	F-Value	*p*-Value	
Model	120.47	9	13.39	219.18	<0.0001	significant
A-Temperature	17.11	1	17.11	280.18	<0.0001	
B-pH	21.78	1	21.78	356.63	<0.0001	
C-Inoculum size	11.76	1	11.76	192.58	<0.0001	
AB	0.0025	1	0.0025	0.0409	0.8454	
AC	0.04	1	0.04	0.655	0.445	
BC	0.0625	1	0.0625	1.02	0.3454	
A^2^	41.12	1	41.12	673.28	<0.0001	
B^2^	8.25	1	8.25	135.13	<0.0001	
C^2^	14.02	1	14.02	229.63	<0.0001	
Residual	0.4275	7	0.0611			
Lack of Fit	0.1675	3	0.0558	0.859	0.531	not significant
Pure Error	0.26	4	0.065			
Source	Sum of Squares	df	Mean Square	F-value	*p*-value	

Note: *p* < 0.01, highly significant; ns, not significant.

**Table 6:** First-order kinetic parameters of prometryn degradation in soil microcosms.

Treatment	k (d − 1)	Half-Life (d)	R^2^	Residual at 30 d (%)
Natural control	0.0119	58.2	0.96	67.6 ± 2.1
ZM-1	0.0564	12.3	0.94	11.4 ± 1.8
ZM-2	0.0492	14.1	0.95	17.6 ± 2.3
ZM-3	0.073	9.5	0.97	7.9 ± 1.5
Synthetic consortium	0.0889	7.8	0.98	3.7 ± 1.2

**Table 7:** Growth parameters of maize after 14 days under different treatments (mean ± SD, *n* = 6).

Treatment	Shoot Length (cm)	Root Length (cm)	Shoot Fresh Weight (g)	Root Fresh Weight (g)
Control (no prometryn)	19.73 ± 0.04	14.28 ± 0.05	5.42 ± 0.07	2.15 ± 0.05
Prometryn alone (80 μg kg^−1^)	7.23 ± 0.06	4.83 ± 0.01	1.30 ± 0.06	0.97 ± 0.05
Prometryn + synthetic consortium	18.92 ± 0.08	13.51 ± 0.09	5.21 ± 0.06	2.08 ± 0.04

## Data Availability

Data Availability Statement: The raw 16S rRNA gene amplicon sequencing data generated in this study are publicly available in the NCBI Sequence Read Archive (SRA) under BioProject accession PRJNA1491339 and SRA study accession SRP71962. All other data supporting the findings of this study are available from the corresponding author upon reasonable request.

## Supplementary Materials

The following supporting information can be downloaded at: https://www.mdpi.com/article/10.3390/microorganisms14092057/s1, Figure S1: LDA score bar plot of stage specific biomarker taxa from LEfSe analysis. Taxa with LDA score > 2.0, *p* < 0.05. Different colors represent taxa enriched at different sampling stages. Note: Please zoom in for better visualization of taxonomic labels.

## Abbreviations

The following abbreviations are used in this manuscript:

ANOVA	Analysis of Variance
BBD	Box–Behnken Design
BSM	Basal Salt Medium
COG	Clusters of Orthologous Groups
HPLC	High Performance Liquid Chromatography
KEGG	Kyoto Encyclopedia of Genes and Genomes
LEfSe	Linear Discriminant Analysis Effect Size
OTU	Operational Taxonomic Unit
NMDS	Non-metric Multidimensional Scaling
RSM	Response Surface Methodology
SRA	Sequence Read Archive
PERMANOVA	Permutational Multivariate Analysis of Variance

## Appendix A

**Table A1:** Correspondence between original sequencing sample IDs and experimental groups.

Majorbio/NCBI Sample ID	Corresponding Group in This Study
0d_1	day 0, biological replicate 1
0d_2	day 0, biological replicate 2
0d_3	day 0, biological replicate 3
7d_1	day 3, biological replicate 1
7d_2	day 3, biological replicate 2
7d_3	day 3, biological replicate 3
14d_1	day 6, biological replicate 1
14d_2	day 6, biological replicate 2
14d_3	day 6, biological replicate 3
21d_1	day 9, biological replicate 1
21d_2	day 9, biological replicate 2
21d_3	day 9, biological replicate 3
24d_1	day 12, biological replicate 1
24d_2	day 12, biological replicate 2
24d_3	day 12, biological replicate 3
