# Supplementary material for: Isolation, Identification of Three Prometryn-Degrading Strains and Their Synthetic Consortium: Degradation Characteristics and Soil Remediation Potential
Source: Microorganisms. 2026 Sep 15;14(9):2057. doi: 10.3390/microorganisms14092057 (PMC13609637; doi:10.3390/microorganisms14092057)
Supplement: Supplementary file 1 [file microorganisms-14-02057-s001.zip › microorganisms-4462166-supplementary.pdf]

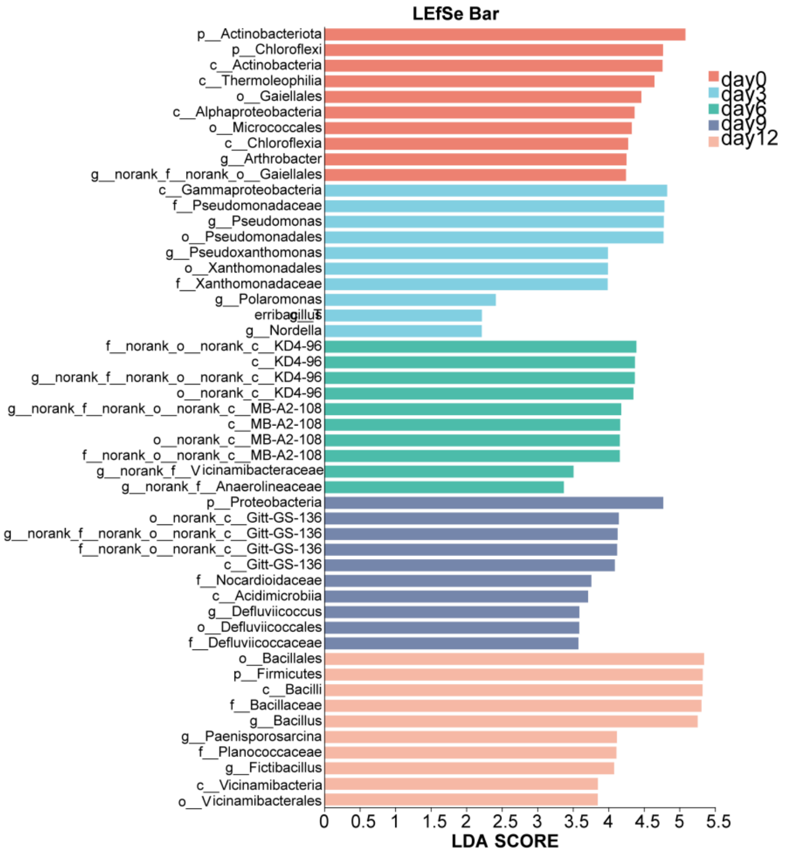

Figure S1. LDA-score bar plot of stage-specific biomarker taxa from LEfSe analysis. Taxa with LDA score > 2.0, $p < 0.05$. Different colors represent taxa enriched at different sampling stages. Note: Please zoom in for better visualization of taxonomic labels.
